# *EO SwimBETTER^®^* Device in Measuring Kinematic and Kinetic Variables: Validity, Reliability, and Sensitivity

**DOI:** 10.3390/jfmk10040428

**Published:** 2025-11-05

**Authors:** Ángel Bastián-Antón, Olga Molinero, Marko Djordjevic, Alfonso Salguero

**Affiliations:** 1 Faculty of Physical Activity and Sports Sciences, University of León, 24195 León, Spain; angelbastiananton@gmail.com; 2 Institute of Biomedicine (IBIOMED), Faculty of Physical Activity and Sports Sciences, University of León, 24195 León, Spain; asalv@unileon.es; 3 University of California San Diego Men and Women Swimming, San Diego, CA 92093, USA; marko@ucsd.edu

**Keywords:** validity, biomechanics, force, *EO SwimBETTER^®^*, feedback, swimming

## Abstract

**Objectives:** The aim of this study was to analyze the utilization of the *EO SwimBETTER*^®^ (*EoLab*, Sydney, Autralia) device for measuring kinetic and kinematic variables during 50 m and 200 m freestyle trials. **Methods:** Ten swimmers (seven males and three females, 20 ± 3.5 years) participated. Each completed three sets of 50 m using *EO SwimBETTER*^®^ on non-consecutive days, with a 200 m test performed during the third session after recovery to complete the *Critical Swim Speed Test* (CSS-T). All tests were conducted at maximal intensity. **Results:** The results showed high reliability for both kinetic and kinematic parameters. Regarding validity, the *EO SwimBETTER*^®^ demonstrated strong agreement with the reference device in measuring stroke frequency (SF). In addition, higher mean force values were found in the 50 m compared with the 200 m trial (Δ% = 8.75%, *p* = 0.099), suggesting sensitivity of the device to different exertion demands. **Conclusions:** Based on these findings, the *EO SwimBETTER*^®^ appears to be a useful and promising tool for monitoring technical and performance-related variables in swimming, although further research is needed.

## 1. Introduction

Swimming is a highly technical sport [1] and is considered a cyclic modality, since between 75 and 85% of the distance covered is achieved through repetitive propulsion action [2,3]. In this context, biomechanics, understood as the science of technique, has established itself as a key area for performance improvement [4,5].

Technical analysis in swimming has evolved significantly in recent decades, moving from primarily qualitative approaches based on videography to the use of advanced technological devices that enable more accurate quantitative analysis. These are usually divided into kinematic studies, which focus on movement (position, speed, acceleration), and kinetic studies, which focus on evaluating the forces involved in propulsion, starts, and turns [5].

Kinetic assessment has become particularly important due to its ability to estimate the mechanical loads acting on the swimmer that directly affect performance. Some authors described swimming technique as a constant struggle against internal and external forces [6]. As early as the 1990s, it was identified that swimming speed depended on the interaction between propulsive and drag forces [7]. Subsequent studies have confirmed that the efficiency with which these forces are applied is a determining factor in performance [8,9,10].

Technological advances have enabled the development of portable, non-invasive devices that can be used in real training conditions. Among these, underwater force meters (m) and inertial measurement units (IMUs) stand out, integrated into tools such as *SmartPaddle*^®^ [11], which allow for the analysis of technical movements without significantly interfering with swimming mechanics [12]. Miniaturization and wireless connectivity have been key factors in the practical implementation of these devices in aquatic environments [13,14].

In this context, *EO SwimBETTER^®^* devices, pressure sensors placed on the palm of the hand that measure the forces generated during underwater traction, have gained popularity [15]. Although still in development, these devices have been shown to meet the technical and ergonomic requirements necessary for use in aquatic environments, as they do not alter the stroke pattern or generate additional resistance [16,17]. Their design enables integrated analysis of propulsive force, stroke length (SL), and swimming speed, as well as the evaluation of the impact of technique on stroke efficiency [18,19]. Recent research concluded that these devices could be a practical tool for evaluating swimming technique and the application of force on the water, both for coaches and researchers [20].

However, despite their increasing adoption, there is still a notable lack of peer-reviewed studies that have rigorously validated the reliability, validity, and sensitivity of *EO SwimBETTER*^®^ in real training conditions. Addressing this gap is essential to support evidence-based use of these devices.

Moreover, understanding how kinematic and kinetic parameters vary under different exertion demands has direct implications for practice: such information can help coaches refine stroke technique, optimize training loads, and provide athletes with objective feedback to improve performance in competitive contexts.

Based on the above, the overall objective of this research was to verify the validity, reliability, and sensitivity of *EO SwimBETTER^®^* devices for recording kinetic and kinematic variables during 50 m and 200 m freestyle tests, exploring their usefulness as a technical assessment tool in real training contexts.

## 2. Materials and Methods

The study design was quantitative, experimental, and prospective.

### 2.1. Participants

Ten swimmers (seven men and three women) belonging to the *León Swimming Club* (CNL, León, Spain) participated in this study, with an average age of 20 ± 3.5 years old. All of them met the inclusion criteria: federative license, training regularly at a competitive level (at least regional). This number represents the final sample, as none of the initially considered participants were excluded due to injury, illness, or refusal to use the *EO SwimBETTER*^®^ device (*EoLab*, Sydney, Australia).

According to previous studies, in research of this type, it is possible to group men and women together in the same analysis, since swimming speed can be evaluated without distinction by sex when seeking to identify technical patterns related to performance [18]. The study was carried out in accordance with the ethical standards of the University of León (Spain) and in accordance with the *World Medical Association* and the *Declaration of Helsinki* [21].

### 2.2. Procedure

This study consisted of the following:

- Initial phase: in which anthropometric data were collected and familiarization sessions with the device were conducted.
- Experimental phase: three sets of 50 m (CSS-T50) using *EO SwimBETTER*^®^ on non-consecutive days (S1, S2, and S3) (see Table 1) at maximum intensity, starting from the water. During these tests, *total time* (TT50), *stroke frequency* (SF), height, body mass, and data from the *EO SwimBETTER^®^* software (v2025) were recorded. In the third session (S3), the CSS-T50 was repeated, followed by a 30 min recovery period. Afterward, a CSS-T200 was performed, both to complete the CSS-T protocol [22] because of the sample characteristics [22,23]. During the CSS-T200, split times were recorded every 50 m, and SF was measured during the second length of each segment. This was performed because factors such as fatigue and pacing strategy can significantly affect kinematic variables like *stroke length* (SL) [24,25], making it essential to monitor them.

The tests were conducted at the swimming facilities of the *Hispánico Sports Center* (León, Spain). Before each session, a standardized warm-up (600–1200 m) was performed, which was customary for the athletes, following the recommendations of Neiva et al. [26]. During the tests, *lane 1* was used for the tests, *lane 2* was left free to avoid waves, and *lane 3* was reserved for warm-up. Two timekeepers timed the trials independently and recorded the technical and time variables from different points in the pool.

### 2.3. Tools

To record anthropometric measurements (height, forearm length, hand width, and hand length), we used a *Stanley 30-085* tape measure with a sensitivity of ±0.1 cm. An *Orbegozo PB 2210 digital scale* with a sensitivity of ±0.1 kg was also used to determine the subject’s mass. These parameters were only entered to configure the instrument and did not form part of the measurements or variables analyzed as study outcomes.

**Figure 1:**
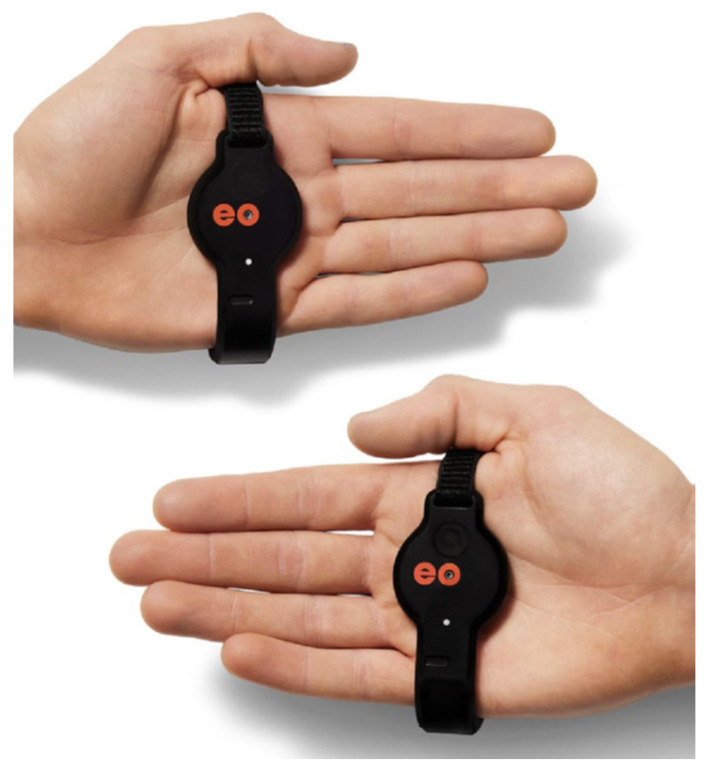
*EO SwimBETTER^®^* sensors.

During testing, *EO SwimBETTER^®^* devices were employed. Each device consists of two independent units attached to the swimmer’s hands. These sensors measure the propulsive force generated by the hands by recording the pressure differences between the palm and the back of the hand [15,27]. In order to analyze the sensitivity of the *EO SwimBETTER^®^* in estimating kinetic variables during the CSS-T, the values of applied force obtained in both distances of the protocol CSS-T50 and CSS-T200 freestyle were compared. This methodological approach follows the recommendations of Lopes et al. and Campos et al. [18,28], who propose the use of this type of device to record data in contexts of variable pace, such as in efforts of different duration or intensity (Figure 1).

In addition, an *Interval 2000 Split/Rate Watch* (NK Sports^®^, Boothwyn, PA, USA), a stopwatch, and a stroke rate monitor with a precision of ±0.01 s and 0.01 s resolution were used to measure SF and time-related parameters. This tool is widely used in professional swimming and rowing settings due to its operational reliability and accuracy in recording split times and SF [29]. The choice of this instrument is based on its practical applicability, ease of use in an aquatic context, and its acceptance among coaches as a real-time timing tool.

### 2.4. Statistical Analysis

We conducted a descriptive analysis by calculating means and standard deviations. To assess normality, we applied the *Shapiro–Wilk test*, which is appropriate for small to moderate samples. Once assumptions were confirmed, we performed parametric tests.

To evaluate reliability, a *one-factor repeated-measures ANOVA* for paired and balanced samples, data collection *sessions* as the independent variable, and a *Student’s t-test* for related samples were performed to compare strength measurements between CSS-T50 and CSS-T200, aiming to assess the sensitivity of the device. Additionally, we calculated the improvement percentage (Δ%) to complete the analysis.

To assess validity, defined as the extent to which the measurements accurately reflect SF, we used *Student’s t-test for related samples*, comparing the recorded values by both devices across the three sessions.

In addition to *p*-values, we calculated effect sizes (Cohen’s *d* for *t-tests*, *partial eta squared* for *ANOVA*) to provide a more comprehensive interpretation of the magnitude of observed differences.

All statistical analyses were conducted using *IBM SPSS Statistics 29.0*, with the level of significance set at *p* < 0.05. Figures were created using *Microsoft Excel 2019*.

## 3. Results

The results were categorized into kinetic and kinematic variables. Regarding the first category, which is less frequently studied and methodologically more complex due to the approaches previously proposed, the outcomes aimed at verifying their reliability are shown in Table 2.

**Figure 2:**
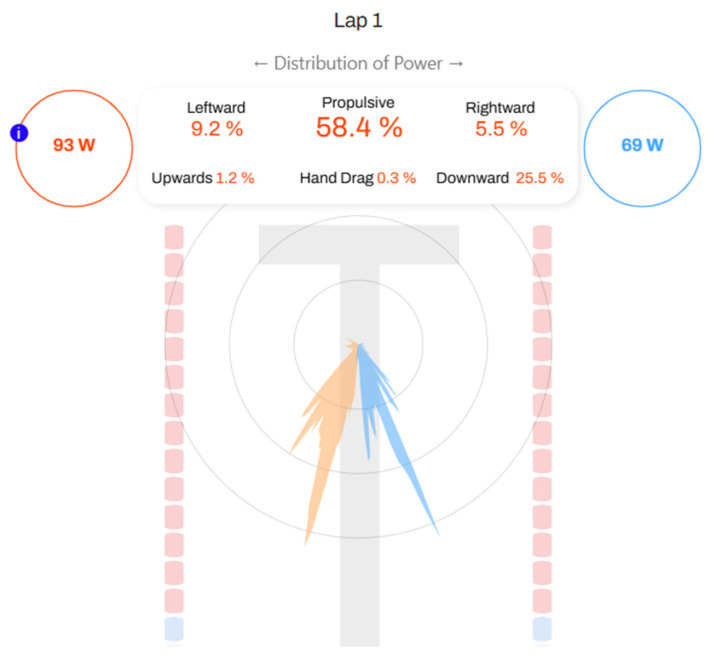
Example of data obtained by the *EO SwimBETTER^®^* web page—kinetic variables. Orange: Left hand. Blue: Right hand.

No significant differences were found between sessions for any of the kinetic parameters, confirming the stability of the measurements across repeated trials. To better illustrate the interpretation of these results, Figure 2 presents an example of PF, VF, and their orientation during the stroke.

This image, provided by the device’s application, offers simple and clear information, serving as an example of the potential applicability and usability of the tool for coaches, technicians, and swimmers. A more comprehensive analysis was performed to evaluate the kinematic variables’ reliability, as well as their sensitivity and validity (Table 3).

The swimming time is decomposed into the main phases of the stroke cycle—glide, pull, and recovery—for both the left and right arms. This breakdown allows for the observation of the relative duration of each phase within the complete movement cycle and provides a detailed view of how the stroke is temporally structured. Together with SF and TT50 values, these data offer the temporal characteristics of swimming technique across sessions. As in the kinetic parameters, the mean values recorded across the three sessions were very similar, and no significant differences were found.

**Figure 3:**
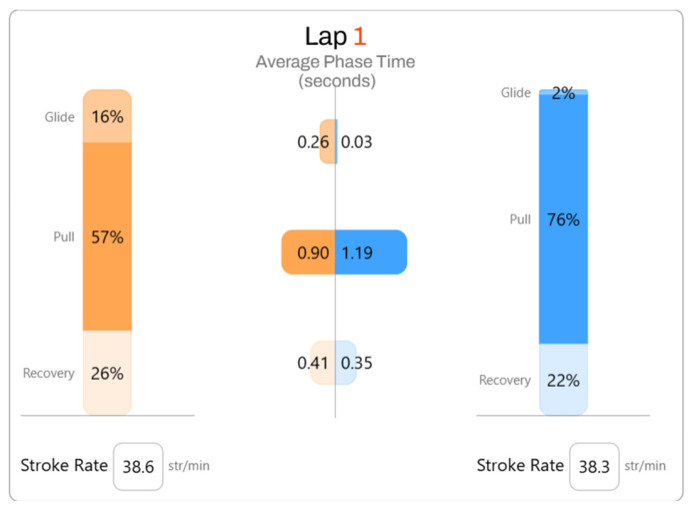
*EO SwimBETTER^®^* web page—kinematic variables. Orange: Left hand. Blue: Right hand.

To illustrate these variables more clearly, Figure 3 provides a visual example that facilitates the analysis of the results for each participant. It complements the quantitative information provided in Table 3 and allows observation of the relative duration of each phase within the stroke cycle.

The validity of the *EO SwimBETTER*^®^ device for measuring FC during the CSS-T50 was evaluated using the *Interval 2000 Split/Rate Watch (NK Sports^®^)* as the reference device. This instrument is widely recognized for kinematic variable analysis, and the comparison between both devices is presented in Table 4. The results show comparable values between both devices, without statistically significant differences in any of the sessions analyzed.

Finally, based on the data obtained on the mean force applied during CSS-T50 (FMT50) and CSS-T200 (FMT200), the percentage difference between the two trials was calculated to assess the magnitude of these variations (Δ% = 8.75%). No statistically significant differences were observed in mean force between CSS-T50 (2.08 ± 0.29 kg) and CSS-T200 (1.92 ± 0.26 kg; *p* = 0.099). However, the calculated effect size (Cohen’s *d* = 0.57) indicated a *moderate* magnitude of difference, suggesting potential practical relevance despite the absence of statistical significance.

## 4. Discussion

The comparative analysis of the data obtained using the *EO SwimBETTER^®^* device during CSS-T50 conducted on different days revealed no significant differences between the measurements taken in the different sessions. These findings demonstrate the reliability of the device in both kinetic and kinematic variables. The stability of these results suggests that *EO SwimBETTER^®^* provides consistent measurements over repeated trials, supporting its applicability for longitudinal monitoring of swimmers’ technical performance.

In relation to both arms’ main forces (FM), the obtained values were lower than those reported by other authors [30,31], probably due to the tools used and characteristics of the sample. Nevertheless, previous research has emphasized the importance of this variable in performance, given its direct relationship with swimming velocity and the anaerobic power of the upper limbs [8,9,11]. Consequently, even with instrument-related differences, the consistent recording of FM across sessions supports the usefulness of *EO SwimBETTER*^®^ for monitoring force-related parameters in real training conditions.

Regarding the kinematic variables, the consistency observed in the data reinforces the potential of the system for technical evaluation [32], particularly for analyzing the relationship between stroke kinematics and performance, especially SL and coordination [33].

SF values demonstrated high reliability across all testing sessions, confirming the accuracy of *EO SwimBETTER^®^* for recording this parameter. This parameter is a key determinant of swimming performance [34], and its optimization involves increasing the number of arm cycles per minute without necessarily increasing the total number of strokes [1]. This relationship is expressed through the *swimming velocity* (SV) formula [3]: SV = SF × SL.

Furthermore, the comparison of SF measured using the *EO SwimBETTER^®^* device and the *Interval 2000 Split/Rate Watch* (*NK Sports*^®^) revealed no significant differences, indicating good agreement between instruments. However, this concurrent validity was assessed exclusively for SF, not for other kinematic variables. It is also important to note that this tool is widely recognized in professional swimming because of its operational reliability and precision (±0.01 s). Therefore, the present results should be interpreted as preliminary but practically relevant evidence of measurement consistency between both tools.

Although the difference in mean force between the 50 m and 200 m trials did not reach statistical significance, the effect size indicated a moderate difference. This suggests that *EO SwimBETTER^®^* was sensitive enough to detect meaningful variations in exertion demands between short- and middle-distance efforts. Variations of up to 8.75% in force values between the two trials further support the sensitivity of the device to changes in effort intensity. From a performance perspective, these differences may have practical relevance for distinguishing between competitive levels [1,33]. Therefore, even without statistical significance, the results highlight the potential of *EO SwimBETTER^®^* to identify relevant biomechanical variations linked to swimming intensity.

The main limitation of the study lies in the small sample size, which restricts the generalizability of the results. Future research should expand the number and diversity of participants to build a more representative database. Moreover, as the present study focused exclusively on freestyle swimming, it would be valuable to explore the kinematics and kinetics of other swimming styles and distances to assess the device’s sensitivity to variations in pace and intensity. Future research could also analyze limb asymmetries and their relationship with propulsive efficiency, given their potential influence on performance. Additionally, comparisons of *EO SwimBETTER^®^* with other measurement systems, such as force gauges or linear encoders, would help to establish stronger concurrent validity for kinetic variables. From a practical perspective, the *EO SwimBETTER*^®^ device may provide coaches and athletes real-time, non-invasive, and easy-to-use solution for monitoring stroke frequency and propulsive force under different exertion demands. This information can support individualized training planning, help monitor fatigue effects, and guide technical corrections aimed at optimizing performance in competitive swimming.

Although no formal assessment of usability, acceptability, or wearability was conducted in the present study, our practical experience suggests that the *EO SwimBETTER*^®^ device can be used comfortably without interfering with swimming technique or performance. Its cost is comparable to other personal training devices currently available on the market. For practical purposes, individualized use is recommended to facilitate calibration, adjustment, and data collection procedures.

## 5. Conclusions

The results of this study support the reliability of the *EO SwimBETTER^®^* device for recording both kinetic (force) and kinematic variables (stroke frequency) performed under real training conditions.

In terms of validity, the *EO SwimBETTER^®^* device showed preliminary but consistent evidence of concurrent validity for stroke frequency when compared with a commonly used reference tool, supporting its potential for accurate kinematic assessment in real training environments.

Regarding sensitivity, the *EO SwimBETTER*^®^ was able to detect differences in mean force between trials (50 m vs. 200 m), with a *moderate* effect size. This suggests that the device can capture meaningful variations in exertion demands, although further research with larger and more diverse samples is required.

Although *EO SwimBETTER*^®^‘s usefulness and usability were not formally assessed, the results and practical observations suggest that it is a feasible and user-friendly tool for technical monitoring and individualized feedback in swimming performance contexts.

## Figures and Tables

**Table 1:** Summary of the direct measurements performed during the study.

Session	Test	Kinetic Variables (Recorded by) (Units)	Kinematic Variables (Recorded by) (Units)
S1	CSS-T50	F_Left_ (EO) (kg) F_Right_ (EO) (kg) FM (EO) (kg)	TT50 (NK) (s) SF (EO, NK) (strokes/min)
S2	CSS-T50	F_Left_ (EO) (kg) F_Right_ (EO) (kg) FM (EO) (kg)	TT50 (NK) (s) SF (EO, NK) (strokes/min)
S3	CSS-T50	F_Left_ (EO) (kg) F_Right_ (EO) (kg) FM (EO) (kg)	TT50 (NK) (s) SF (EO, NK) (strokes/min)
	CSS-T200	F_Left_ (EO) (kg) F_Right_ (EO) (kg) FM (EO) (kg)	TT50 (NK) (s) SF (EO, NK) (strokes/min)

S = Data collection session; F = Mean Force; FM = Both Arms Mean Force; TT50 = Mean time recorded by the two evaluators; SF = Stroke frequency; Left = Left Arm; Right = Right Arm; EO = Recorded by the *EO SwimBETTER^®^*; NK = Recorded by the *Interval 2000 Split/Rate Watch device (NK Sports^®^)*.

**Table 2:** Descriptive and comparative analysis of kinetic variables across different data collection sessions in CSS-T50.

Kinetic Variables (Units)	Session	n	M ± Sd	*p*
F_Left_ (kg)	S1	10	2.03 ± 0.38	0.986
	S2	8	2.06 ± 0.36	
	S3	9	2.03 ± 0.36	
F_Right_ (kg)	S1	10	2.07 ± 0.36	0.928
	S2	8	2.06 ± 0.35	
	S3	9	2.01 ± 0.38	
FM (kg)	S1	10	2.05 ± 0.33	0.962
	S2	8	2.06 ± 0.29	
	S3	9	2.02 ± 0.33	
PF (%)	S1	10	42.20 ± 11.88	0.817
	S2	8	43.20 ± 12.01	
	S3	9	45.49 ± 10.37	
VF_Left_ (%)	S1	10	40.73 ± 16.60	0.813
	S2	8	38.53 ± 15.92	
	S3	9	36.06 ± 14.55	
VF_Right_ (%)	S1	10	46.11 ± 12.21	0.437
	S2	8	40.54 ± 13.23	
	S3	9	37.86 ± 16.42	

M = Mean; Sd = Standard Deviation; *p* = Level of significance; S = Data collection session; F = Mean Force; FM = Both Arms Mean Force; PF = Propulsive Force; VF = Vertical Force directed downward; Left = Left Arm; Right = Right Arm.

**Table 3:** Descriptive and comparative analysis of kinematic variables across different data collection sessions in CSS-T50.

Kinematic Variable	Session	n	M ± Sd (s)	*p*
TT50 (s)	S1	10	30.73 ± 3.06	0.972
	S2	10	31.01 ± 3.04	
	S3	10	31.01 ± 3.08	
SF_NK_ (strokes/min)	S1	10	56.03 ± 4.31	0.780
	S2	10	56.33 ± 5.00	
	S3	10	54.90 ± 4.88	
SF_EO_ (strokes/min)	S1	10	55.66 ± 3.70	0.785
	S2	8	56.06 ± 3.56	
	S3	9	54.81 ± 4.09	
GLIDE_Left_ (%)	S1	10	19.55 ± 9.33	0.226
	S2	8	18.75 ± 8.83	
	S3	9	13.61 ± 3.72	
GLIDE_Right_ (%)	S1	10	17.50 ± 6.84	0.938
	S2	8	16.88 ± 4.29	
	S3	9	18.00 ± 7.53	
PULL_Left_ (%)	S1	10	49. 65 ± 12.69	0.883
	S2	8	51.25 ± 14.33	
	S3	9	52.33 ± 7.34	
PULL_Right_ (%)	S1	10	58.25 ± 8.41	0.655
	S2	8	55.38 ± 5.15	
	S3	9	58.28 ± 7.81	
REC_Left_ (%)	S1	10	30.75 ± 5.36	0.386
	S2	8	30.06 ± 6.34	
	S3	9	33.94 ± 6.86	
REC_Right_(%)	S1	9	25.39 ± 4.14	0.240
	S2	8	27.81 ± 1.60	
	S3	7	26.14 ± 1.86	

M = Mean; Sd = Standard deviation; s = seconds; *p* = Level of significance; S = Data collection session; TT50 = Mean time recorded by the two evaluators; SF = Stroke frequency; GLIDE = Time spent in the glide phase; PULL = Time spent in the pull phase; REC = Time spent in the recovery phase; NK = Recorded by the *Interval 2000 Split/Rate Watch device (NK Sports^®^)*; EO = Recorded by the *EO SwimBETTER^®^*; Left = Left Arm; Right = Right Arm.

**Table 4:** Descriptive and comparative analysis of SF in CSS-T50.

Session	SF_NK_CSS-T50 (Strokes/min)	SF_EO_CSS-T50 (Strokes/min)	*p*
	M ± Sd	M ± Sd	
S1	56.03 ± 4.31	55.66 ± 3.70	0.549
S2	56.33 ± 5.00	56.06 ± 3.56	0.290
S3	54.90 ± 4.88	54.81 ± 4.01	0.668

M = Mean; Sd = Standard deviation; *p* = Level of significance; S = Data collection session; SF = Stroke Frequency; NK = Recorded by the *Interval 2000 Split/Rate Watch device (NK Sports^®^)*; EO = Recorded by the *EO SwimBETTER^®^*.

## Data Availability

The data presented in this study are not publicly available due to privacy and ethical restrictions, but may be made available upon reasonable request from the corresponding author, subject to approval by the relevant ethics committee.

## Abbreviations

The following abbreviations are used in this manuscript:

CSS-T	Critical Swim Speed Test
CSS-T200	200 m Protocol Critical Swim Speed Test
CSS-T50	50 m Protocol Critical Swim Speed Test
EO	Recorded by the *EO SwimBETTER^®^*
F	Mean Force
FM	Both Arms Mean Force
FMT200	Mean force during CSS-T200
FMT50	Mean force during CSS-T50
GLIDE	Time spent in glide phase
kg	kilos
m	meters
M	Mean
NK	Recorded by the *Interval 2000 Split/Rate Watch device (NK Sports^®^)*
p	Level of significance
PF	Propulsive Force
PULL	Time spent in the pull phase
REC	Time spent in the recovery phase
S	Data collection session (S1, S2 and S3)
s	seconds
Sd	Standard Deviation
SF	Stroke frequency
SL	Stroke length
SV	Swimming velocity
TT50	Mean time recorded by the two evaluators
VF	Vertical Force directed downward
